# Appraising Evidence-Based Mental Health and Psychosocial Support (MHPSS) Guidelines—PART II: A Content Analysis with Implications for Disaster Risk Reduction

**DOI:** 10.3390/ijerph19137798

**Published:** 2022-06-25

**Authors:** Michel Dückers, Wera van Hoof, Andrea Willems, Hans te Brake

**Affiliations:** 1 ARQ Centre of Expertise for the Impact of Disasters and Crises, 1112 XE Diemen, The Netherlands; w.van.hoof@arq.org (W.v.H.); a.willems@arq.org (A.W.); h.te.brake@arq.org (H.t.B.); 2 Nivel-Netherlands Institute for Health Services Research, 3513 CR Utrecht, The Netherlands; 3 Faculty of Behavioural and Social Sciences, University of Groningen, 9712 TS Groningen, The Netherlands

**Keywords:** guidelines, quality, mental health and psychosocial support (MHPSS), DRR

## Abstract

High quality mental health and psychosocial support (MHPSS) guidelines are indispensable for policy and practice to address the mental health consequences of disasters. This contribution complements a review that assessed the methodological quality of 13 MHPSS guidelines. We analyzed the content of the four highest-ranking guidelines and explored implications for disaster risk reduction (DRR). A qualitative explorative thematic analysis was conducted. The four guidelines proved largely similar, overlapping or at least complementary in their MHPSS definitions, stated purpose of the guidelines, user and target groups, terminology, and models used. Many recommended MHPSS measures and interventions were found in all of the guidelines and could be assigned to five categories: basic relief, information provision, emotional and social support, practical support, and health care. The guidelines stress the importance of monitoring needs and problems, evaluating the effect of service delivery, deliberate implementation and preparation, and investments in proper conditions and effective coordination across professions, agencies, and sectors. The MHPSS knowledge base embedded in the guidelines is comprehensive, coherent, and sufficiently universal to serve as the “overarching framework” considered missing yet vital for the integration of MHPSS approaches in DRR. Although application contexts differ geographically, this common ground should allow policymakers and practitioners globally to plan, implement, and evaluate MHPSS actions contributing to DRR, ideally together with target groups.

## 1. Introduction

Disasters and humanitarian crises can have a tremendous impact on the mental health and psychosocial well-being of affected populations [1,2,3,4,5,6,7,8]. To adequately respond to mental health risks and problems, governments and partners at different levels, including governmental and non-governmental organizations and citizens, need to engage and collaborate on behalf of the planning and implementation of mental health and psychosocial support (MHPSS) in different stages of the disaster life cycle [9,10,11,12,13,14,15,16,17]. MHPSS has been defined as “any type of local or outside support that aims to protect or promote psychosocial well-being and/or prevent or treat mental disorder” [18] (p. 1).

Several authors have emphasized the importance of integrating MHPSS with the field of disaster risk reduction (DRR) [19,20]. DRR, in its broad sense, is “aimed at preventing new and reducing existing disaster risk and managing residual risk, all of which contribute to strengthening resilience and therefore to the achievement of sustainable development” [21]. Approaching risk through an MHPSS lens gives DRR a specific focus, namely of risk reduction linked to the well-being and mental health of individuals or groups affected by possible and actual disasters. The MHPSS field offers perspectives and approaches that can amplify the impact of DRR activities in this respect [19]. At the same time, integrating MHPSS guidance into DRR policy and practice has been shown to be rather challenging [20]. Hereafter, before outlining the study objective, we will go deeper into some of these challenges together with the type of process that might help to overcome them.

### 1.1. Challenges in Integrating MHPSS with DRR Programming

Gray and colleagues recently reviewed available literature from databases and relevant documentation and project descriptions from MHPSS actors to gain insight into the integration of MHPSS and DRR [20]. A few key challenges described in the analysis can be summarized as:

- The material points at a lack of “consensus or overarching framework”. No “definition or consensus-based model for discussing MHPSS components of DRR” could be identified. MHPSS and DRR are typically discussed in isolation, without combined narrative (likewise for the findings of a literature review exploring the cross-section of MHPSS principles and crisis management [10]).
- There is limited consensus or understanding of “what activities constitute the integration of MHPSS and DRR”. Similarly, there is no clear consensus on “MHPSS placement in DRR policy or guidance on best practices for working with various stakeholders to integrate MHPSS with existing DRR programming”.
- Evidence-based or consensus-driven definitions and guidelines for integrating MHPSS into DRR programming are necessary for an effective widespread implementation [20].

### 1.2. MHPSS Knowledge Development and Uptake on Behalf of Disaster Risk Reduction

#### 1.2.1. Disaster Risk Reduction: Two Focal Areas

What the three challenges listed above have in common is that they mirror shortcomings in consensus among stakeholders on what DRR requires from an MHPSS perspective. These stakeholders belong to different domains—science, policy, and practice—that, preferably, work jointly to facilitate a mental-health-risk-oriented disaster policy plan, as well as its implementation. It is important to establish and maintain a reciprocal exchange across the three domains. Généreux and colleagues described a series of sequential steps in the science domain (problem, research, and knowledge) that ideally benefit from input from the domain’s policy and practice, and are succeeded by a second series of sequential steps in the overlapping area of the latter domains (transfer, adoption, and diffusion) [22]. Whereas the first series results in research-informed knowledge development, the second series deals with its practical application and dissemination.

**Figure 1:**
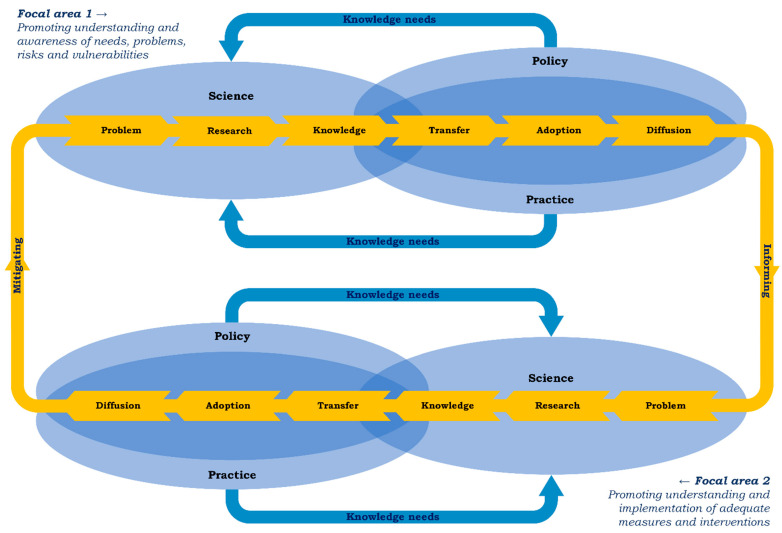
Disaster risk reduction through knowledge development and application. Shown here are two focal areas with series of steps that ideally influence each other. Knowledge about needs, problems, risks, and vulnerabilities, generated and shared in Focal area 1 can inform the activities in Focal area 2 to mitigate issues of concern. The exchange between stakeholders from science, policy, and practice is important to produce knowledge and tools (co-learning) based on scientific methods (verified, tested) and relevant for policy and practice (support, ownership).

In principle, the steps are applicable to two focal areas within MHPSS knowledge development and uptake on behalf of DRR: (1) understanding disaster mental health risks, problems, and contributing factors in general and for vulnerable populations in particular; (2) understanding effective and efficient responses and how to implement them in practice by a variety of stakeholders. One could argue that through the interaction between stakeholders representing each of the three domains, along the entire chain of steps, progress can be realized under each of the focal areas (see Figure 1).

The first focal area can be approached through (rapid) needs assessments, vulnerability assessments, and disaster health research [23,24,25,26,27,28,29]. The growing international epidemiological literature is an invaluable starting point to make an initial estimate of probable mental health needs, problems, and vulnerabilities in different disaster stages of a hypothetical scenario or an actual unfolding event. Input from policy and practice can add focus to the design and implementation of monitoring activities post disaster, enhancing ownership among recipients of the research-based knowledge, and therefore, its likeliness of further practical utilization and dissemination (the upper part of Figure 1).

Concerning the second focal area, this line of reasoning is equally relevant to the development and, ultimately, the implementation of solutions for the benefit of DRR. More specifically, the steps delineate a path to produce and apply promising MHPSS perspectives and approaches to reduce mental health risks (the lower part of Figure 1).

We assume that DRR is served at maximum when the series of steps per focal area influence each other: knowledge from Focal area 1 *informs* the process under Focal area 2 that, when knowledge is applied, *mitigates* the issues determined in Focal area 1 [30]. Another defendable assumption is that the dialogue should be shaped along community engagement standards concerning participation, empowerment and ownership, inclusion, two-way communication, adaptability, and localization and building on local capacity [31].

#### 1.2.2. Zooming in on the Second Focal Area: MHPSS Knowledge Captured in Guidelines

When it comes to solutions, the second focal area, an indispensable contemporary source of MHPSS knowledge, consists of various guidelines that have been developed during the last decades with the intention of combining scientific evidence and insights from policy and practice. As such, MHPSS guidelines, which commonly include findings from literature reviews, desk research, expert interviews and group discussions, and sometimes, through Delphi methods or surveys [32,33,34,35,36,37,38,39], fit well within the logic of Figure 1. The guidelines resulting from dialogue contain a variety of models, theories, guiding principles, and recommended (or discouraged) measures and interventions. In this contribution, we seek to test the idea that the content of existing guidelines might play a meaningful role in the integration of MHPSS into DRR. An analysis of guidelines might point to areas and topics to make the integration more explicit and help us move beyond the tendency to treat MHPSS and DRR as separate topics.

### 1.3. Study Focus

The study aim is to appraise how DRR can benefit from the content of MHPSS guidelines. For this purpose, we will analyze a set of four guidelines that received the highest methodological quality scores in an accompanying review [40]. We formulated three objectives for our comparative analysis: firstly, to explore basic characteristics of the guidelines such as the intended application context, MHPSS definitions, objectives, and user and target groups; secondly, to disentangle the nature of the guidance by giving an overview of terminology, models, and recommended measures and interventions; and thirdly, to examine to what extent implementation or preparedness conditions are considered.

## 2. Materials and Methods

### 2.1. Selection of Guidelines

Te Brake and colleagues conducted a comparative analysis of the methodological quality of available MHPSS guidelines [41]. A systematic literature search was conducted in seven databases (including PsycInfo, Medline, Embase, Cochrane Library, PILOTS, Sociological Abstracts, and Web of Science) to collect relevant publications from 2007 up to June 2021 that use an international guideline, manual, framework, review, toolkit, or implementation plan concerning MHPSS in disaster response for analysis, comparison, or evaluation. The resulting articles were screened based on title and abstract by independent researchers. The search included ‘grey literature’ from the E.U. project Operationalizing Psychosocial Support in Crisis (OPSIC) [12,38], the growing online platform of mhpss.net, and ReliefWeb, a humanitarian information service provided by the United Nations Office for the Coordination of Humanitarian Affairs (OCHA) [41,42]. Documents were assessed using predefined inclusion criteria and eventually 13 documents (a) met the guideline definition formulated by the Appraisal of Guidelines for Research and Evaluation (AGREE), and (b) focused on improving MHPSS in disaster/emergency/humanitarian settings. Each guideline was assessed by 3–5 raters using the AGREE-HS instrument covering the following categories: topic; participants; methods; recommendations; and implementability [43,44]. The overall quality level of the 13 guidelines varied considerably. The average AGREE-HS score was 45.4, with scores ranging between 21.3 and 67.6 on a scale from 0 to 100 [40]. For the current analysis, we included MHPSS guidelines scoring 50 or higher. The following guidelines met this criterion (short title; publication year; AGREE-HS score):

- Inter-Agency Standing Committee (IASC guidelines; 2007; 63.6) [18].
- Dutch multi-disciplinary guidelines (Dutch or Impact guidelines; 2014; 67.6) [45].
- Operationalising Psychosocial Support in Crises (OPSIC guidelines; 2016; 56.8) [38].
- International Committee of the Red Cross (Red Cross guidelines; 2018; 60.8) [46].

### 2.2. Data Extraction

To structure the data extraction, we defined a few themes. Information was extracted on the socioeconomic context of the guidelines, MHPSS definitions, user groups, target groups, terminology (linked to Focal areas 1 and 2 in Figure 1), and models (e.g., theoretical concepts, core ideas, principles). To extract information on recommended measures and interventions, an existing categorization scheme for disaster service delivery was used: “basic aid” (i.e., shelter, safety, food, drinking water, first aid, and medication), “information” (i.e., about what has happened, about the fate of loved ones, about normal reactions), “social and emotional support” (i.e., comfort, a listening ear, recognition of grief, compassion, social acknowledgment), “practical help” (i.e., legal and financial issues, household), and “health care” (i.e., adequate detection and treatment of mental health risks and problems, ranging from prevention to specialized treatment) [26,45,47]. Furthermore, we looked for information in the guidelines on monitoring and evaluation [9,45,47,48] and conditions for guideline implementation or (preparedness for) the planning and delivery of MHPSS (e.g., resources, coordination, training).

### 2.3. Analysis

The guidelines were coded using the qualitative software program MAXQDA, a software package for qualitative and mixed methods data analysis (2022, Verbi, Berlin). Four researchers with expertise in MHPSS guidelines were involved in the analysis. Two researchers (W.v.H. and A.W.) analyzed the four guidelines independently. The data analysis was based on a partly theoretical, partly inductive approach [49] (pre-structured as described above), but at the same time, the content of the guidelines was examined exploratively to avoid any prior assumptions of the outcome. During this process, the differences in coding were contentiously reviewed and discussed by the two researchers until consensus was reached. The emerging coding framework/tree represented the substantive and recurring topics identified. Preliminary findings were discussed with the other researchers (M.D. and H.t.B.) in every step of the process as we strived for intersubjectivity of interpretation [49]. After the coding process, the two researchers who analyzed the guidelines independently, reviewed all emerged segments in detail. The topical categorization of codes was used to structure the outcomes from the analysis in the next section.

## 3. Results

### 3.1. Characteristics of the Guidelines

#### 3.1.1. Context

The four guidelines were designed for different MHPSS settings. The IASC guidelines (2007) are oriented primarily on low- and middle-income countries, while the overall framework is considered applicable to disasters in high-income countries. The OPSIC guidelines (2016) make a clear distinction in recommendations for different phases of the disaster timeline and reflect knowledge from both high-income and low-income settings. The ICRC guidelines (2018) emphasize situations of armed conflict and violence. The first edition of the Impact guidelines was translated into English [34] and scored for relevance and applicability by international experts, predominantly from high-income countries (under the umbrella of the EU-project EUTOPA) [37]. In this study, we analyzed the second edition (2014), written in Dutch and tailored to the context of the Netherlands, a high-income country.

#### 3.1.2. Definitions, Purpose, User Groups, and Target Groups

Table 1 gives an overview of the guidelines’ definition of MHPSS, the stated purpose of the guidelines, and the user and target groups. While there is no unifying definition, the guidelines use similar language to define MHPSS, always encompassing the promotion of mental health and well-being (best effort obligation), and sometimes directly reducing, even avoiding, psychopathology (result obligation).

The guidelines serve comparable purposes: to provide information and tools to help structure the planning and delivery of MHPSS in crisis situations, with more or less explicit guidance for different settings and phases. Most guidelines focus on decision-makers, crisis managers, and practitioners like mental health professionals and aid workers (including volunteers). The target groups of the guidelines—MHPSS beneficiaries—are rather comprehensive. They commonly recognize vulnerable populations such as women, children, people with existing mental health issues, refugees, and people with other disadvantageous conditions as target groups. The Red Cross guidelines (chapters 2 to 7) and the OPSIC guidelines (see the action sheets in “Part II”) use the distinction in target groups to structure the guidance for the user groups.

### 3.2. Nature of the Guidance

#### 3.2.1. Emphasis in Terminology

The number of pages and the layout of the guidelines differ substantially, with page numbers varying from 88 (Impact) to 112 (Red Cross) to 190 (IASC) to 423 (OPSIC). It is not meaningful to compare the frequency of terms *between* the guidelines, but *within* the documents, it helps to understand the relative attention given to themes and topics.

Table 2 gives an overview of terminology used in the four guidelines, clustered along the focal areas of Figure 1. Differences in accents for Focal area 1 can be seen across the documents. Risks, problems, and disorders are dominant themes in the IASC guidelines. The Impact guidelines place an emphasis on problems, needs, and resiliency. Resilience is among the most frequently counted terms of the OPSIC guidelines, together with risks and needs. The OPSIC guidelines place more focus on trauma and post-traumatic stress disorder (PTSD) than the other ones. The terms observed most often in the Red Cross guidelines are needs and, to a lesser extent, disorders and problems. Regardless of differences in accent, Focal area 1 terms can be found multiple times in each of the guidelines.

This is also the case with Focal area 2, especially in the IASC, Red Cross, and OPSIC guidelines. Again, the relative weight of themes is not the same in the guidelines. The IASC guidelines give more attention to assessment, training, and resources, followed by coordination. In the Impact guidelines, “promoting natural recovery and utilization of natural capacity” and “detection and responding to current needs and (mental health) problems” are part of the objective of MHPSS. Nevertheless, the guideline terminology is focused more on evaluation and preparation. The terms observed the most in the OPSIC and Red Cross guidelines are linked to assessment, resources, and training. Preparedness and prevention are dominant themes in the OPSIC guidelines as well.

Table 2 contains additional themes that are meaningful for both focal areas because potential disaster-related MHPSS issues of concern and solutions are likely to depend on circumstances and context that can vary between phases and cultures. Context and circumstances are mentioned repeatedly in all of the guidelines. The Impact, OPSIC (“Part II”), and IASC guidelines are clearly more explicit about phases than the Red Cross guidelines. IASC and OPSIC give more attention to a culturally appropriate response and human rights, and ethics is clearly present in the OPSIC guidelines. The Impact guidelines, however, do not pay explicit attention to ethical aspects of MHPSS and human rights at all.

#### 3.2.2. Models

**Figure 2:**
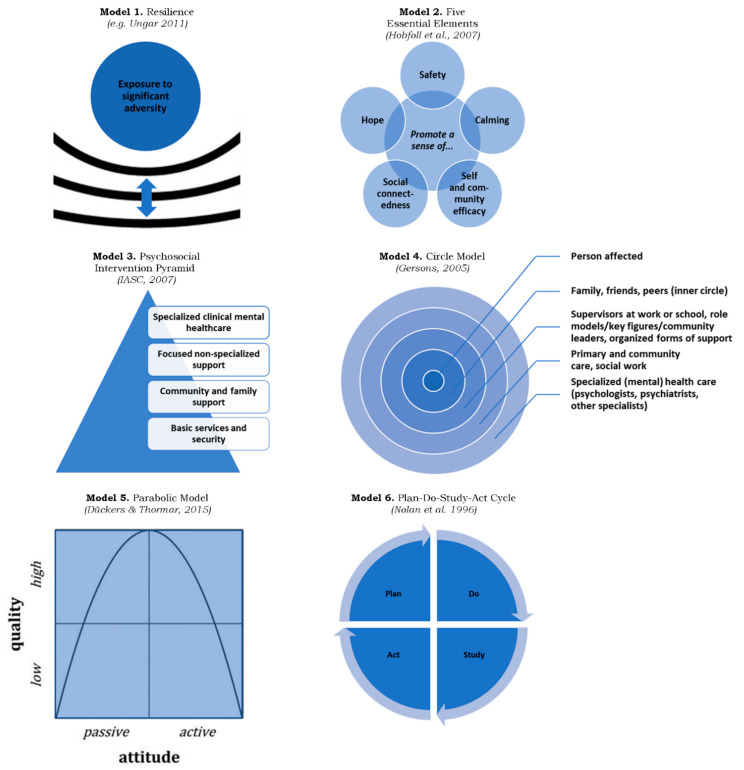
Six Models (Ungar 2011 [51], Hobfoll et al. 2007 [52], IASC 2007 [17], Gersons 2005 [55], Dückers & Thormar 2015 [47], Nolan et al. 1996 [56]).

Where the terminology gives an idea of the themes addressed in the guidelines, the documents contain several models that are helpful to understand what the guidelines are telling their user groups. Six of these models identified are shown in Figure 2.

The first model, resilience, can be defined or conceptualized in many ways. Some guidelines are explicit in their description of what resilience entails. In the glossary of the OPSIC guidelines, a DRR, as well as an MHPSS definition, is included. The first one comes from the United Nations Institute for Disaster Risk Reduction and includes the ability to resist, absorb, accommodate, adapt to, transform, and recover from the effects of a hazard, including the “preservation and restoration of essential basic structures and functions” [50]. The second one, closer to the language of MHPSS, was taken from Ungar: “In the context of exposure to significant adversity, resilience is both the capacity of individuals to navigate their way to the psychological, social, cultural, and physical resources that sustain their wellbeing, and their capacity individually and collectively to negotiate for these resources to be provided in culturally meaningful ways” [51] (p. 14).

Next, Hobfoll and colleagues suggest that supportive services towards target groups should promote a sense of safety, calming, self and community efficacy, social connectedness, and hope [52,53,54]. The “five essential elements” (Model 2) can be seen as supportive to resilience and are included in the Impact and the OPSIC guidelines.

The “psychosocial intervention pyramid” makes a distinction between the different priorities, risks, and needs of affected individuals. In Figure 2, this is represented in the shape of a pyramid with a broad base and a narrow top (Model 3). In the top of the pyramid, we find specialized clinical mental health care, which is most important for treatment needs of a smaller proportion of the population. Downwards, we find other types of services that are increasingly of more general need and can be provided by people’s communities, families, friends, and other social networks. The pyramid was described in the IASC guidelines and incorporated later in the OPSIC guidelines.

The “circle model”, abstracted by Gersons, follows an analogous line of reasoning [55]. At the center of the model, we find the more or less resilient individual. This individual is surrounded by circles with other people who can provide support and care in a stepwise manner (Model 4 in Figure 2). The first circle consists of a person’s own close social network. The second circle is still personal, but exceeds the inner circle and covers community actors, the working environment, and other social groups. The third circle involves basic health care professionals (primary and community health care) and, if health problems are still not sufficiently addressed here, there is a complementary fourth circle of specialized health care. The circle model, with its gradual progression of support intensity depending on the needs and (lack of) resilience of affected individuals, is one of the key models in the Impact guidelines.

The fifth model, given a place in the Impact and the OPSIC guidelines, is the “parabolic model”, conceptualized by Dückers and Thormar [47]. The model combines two dimensions. The quality of MHPSS (*y*-axis) can be expressed in scores, assigned to criteria like need-centeredness, effectiveness, safety, timeliness, efficiency, and equity, which are proposed to be related to the “attitude” (*x*-axis), more passive or active, towards affected target groups. Preferably, the MHPSS approach is found in the “high quality” middle zone of the attitude-axis. Extremely passive or active “low quality” approaches are to be avoided. High quality is associated with responsible behavior, avoiding waste and harm, and not overestimating (too passive) or underestimating (too active) resilience. The quality threshold should be monitored [47].

The plan-do-study-act cycle, originating from quality management literature [56,57,58], prescribes that the planning and implementation of MHPSS approaches should be accompanied by a timely check to verify whether expected effects or results occur, or whether the approach is not too passive or active [47]. This sixth model is included explicitly in the Impact guidelines. In the other guidelines, the model is integrated in the guidance more implicitly, for example: “The quality threshold is to be guarded. Programme managers and service providers who check/monitor whether their plans and expectations regarding a diversity of individuals or communities come true, bring a safety valve into the programme” (OPSIC guidelines); “Monitoring and evaluation involves an ongoing, systematic process of recording, collecting, measuring, analyzing and transmitting information in order to identify areas for improvement and, ultimately, to achieve defined objectives more efficiently” (Red Cross guidelines); “Conduct regular assessments of the accessibility and quality of mental health care” (IASC guidelines).

#### 3.2.3. Measures and Interventions

In Table 3, a summary is given of recommended measures and interventions found in the various guidelines. The information in the table is a simplified overview, structured along the five MHPSS categories as distinguished in the Impact guidelines. The categories basic aid, information, emotional and social support, and practical support are considered relevant regardless of their relation to the (mental) health impact of disasters and crises. They can be justified from a humanitarian perspective. Furthermore, when approached from a health care perspective (the fifth category), they can be interpreted as potentially preventive or protective strategies when respectful towards risks, needs, and problems.

All the guidelines, despite considerable variation in level of detail, contain measures and interventions fitting multiple MHPSS categories. Table 3 gives an impression of the level of overlap or consensus. The number of items mentioned in only one of the four guidelines is small (e.g., activating a telephone helpline or compensation), and many of the items are included in all four of the guidelines (e.g., food and water, psychological first aid, ensuring access to specialized mental health care). Where the guidelines primarily contain *recommended* activities, psychological debriefing—a scrutinized preventive measure for the development of PTSD symptomatology—is explicitly *not recommended* in the guidelines. There is evidence that this intervention can lead to worsening instead of the reduction of psychological complaints, although this evidence is also criticized [59,60]. The guidelines we analyzed reflect a precautionary principle—they are modest in recommending interventions to prevent or even predict psychopathology. Screening, for instance, can give an indication of current mental health problems or concerns, but it remains unpredictable how they will further evolve.

When guidelines provide recommendations for the diagnosis and treatment of stress-related disorders, anxiety disorders, mood disorders, substance use, and medically unexplained symptoms, these are primarily formulated in general terms with reference to other evidence-based guidelines for general practice and specialized mental health.

What complicates matters is that guidelines use umbrella terms to describe measures and interventions. Consequently, it is not always possible to determine whether it is allowed to interpret synonyms, or even homonyms, in an identical way. One example is how coordinated service centers are described. The term “one-stop shop” is applied in Impact and OPSIC guidelines. The Impact guidelines describe the one-stop shop as “a physical or online information and service centre” and “a fixed point of departure for contact with the affected, enabling to track needs and problems, promote contact between the affected (peer support), and assist with legal issues in the aftermath of the event”; a place where multiple categories of services from different providers are brought together and are made accessible. The OPSIC guidelines distinguish between the short and long term. “Reception centre” is reserved for the acute phase (non-injured, family, and friends). Several synonyms for long-term one-stop shop are used such as “humanitarian assistance centre”, “community centre”, “long-term shelter”, “evacuation centre” or “coordination points for long-term care and support”. The IASC and Red Cross guidelines consider protective shelters and “collaborative service/collaboration” important, but contain no explicit recommendations to establish one-stop shops or service centers for the short or long term. In another example, the IASC and OPSIC guidelines speak of “relaxation” or “relaxing” as a “stress management technique” or contributing to calmness. This can be considered an element of psychological first aid, yet psychological first aid covers more elements, and these can also be approached as separate interventions. In a third example, all the guidelines recommend a form of screening for risk and vulnerability factors. In the OPSIC guidelines, an additional differentiation is made: “screening (through use of generic forms and self-report measures)”, “clinical review if indicated (e.g., very high arousal, behavioural disturbance, cognitive impairment)” and “comprehensive mental health assessment (for symptomatology and specific syndromes)”.

#### 3.2.4. Monitoring and Evaluation

Monitoring and evaluation are considered an overarching recommended set of activities in all the guidelines, transcending the five categories in Table 3. It involves gaining an idea of needs, problems, risks, and vulnerabilities of target groups, verifying whether measures and interventions are producing the desired result, and making adjustments if necessary (the essence of the plan-do-study-act model). In general, all the guidelines contain recommendations fitting this purpose. To gain insight into immediate or probable issues, the guidelines encourage registration (not given attention in the Red Cross guidelines), screening, and needs assessments. The proposed methods range from generic forms (not mentioned in Red Cross guidelines), standardized scales (e.g., distress, functioning, and coping), and interviews.

The IASC guidelines state that it is essential to understand how to support affected populations to more constructively address MHPSS needs. Assessments, for this reason, “must also be part of an ongoing process of collecting and analyzing data in collaboration with key stakeholders, especially the affected community, for the purposes of improved programming”. Moreover, in the IASC guidelines, “participatory assessment” is described as “the first step in a dialogue with affected populations, which, if done well, not only provides information but may also help people to take control of their situation by collaboratively identifying problems, resources and potential solutions”.

Monitoring and evaluation can be divided into collaborative activities, beneficial to Focal areas 1 and 2 in Figure 1, that, in combination, might indeed result in MHPSS practices where activities are based on co-owned knowledge on what is necessary, followed by a check, a moment to verify whether observations are satisfactory. In order to guide the check, some of the guidelines suggest specific MHPSS output or outcomes to take into account (Red Cross guidelines), or to collect information on the structure, process, and outcome of MHPSS provision (OPSIC guidelines), or on the realization of particular criteria e.g., effectiveness, efficiency, need-centeredness (Impact guidelines). Guidelines rarely recommend specific instruments or methods to collect evaluation data. The Impact guidelines encourage the development of evaluation instruments, ideally built upon existing instruments in collaboration between service providers and researchers to benefit from expertise from science and practice, and together with responsible organizations and governments to ensure that lessons are translated into improvement efforts.

#### 3.2.5. Implementation and Preparedness

All the guidelines address the practical adoption of their content, as reflected in the usage of terms like implementation, preparedness, and training (Table 2).

The IASC guidelines are detailed in their recommendations for implementation and preparedness. The preparedness guidance is worked out in a “matrix of interventions”, divided into three parts: common functions across domains (i.e., coordination, assessment, monitoring and evaluation, protection and human rights standards, human resources); core MHPSS domains (i.e., community mobilization and support, health services, education, dissemination of information); and social considerations in sectoral domain (i.e., food security and nutrition, shelter and site planning, water and sanitation). The vocabulary of the guidance also included mapping, awareness raising, planning, and capacity building.

The Red Cross guidelines approach implementation differently. These guidelines are structured with recommendations for distinctive target groups as listed in Table 1. A section is added with “main challenges for programme implementation” per target group. Generically, the guidelines recommend to “design and implement” MHPSS “programmes” using a ”multidisciplinary approach”, “integrated in mental health care” and, preferably, with “long-term support” and “follow up”.

In the OPSIC guidelines, implementation recommendations are integrated in action sheets for universal topics such as ethical, gender, and cultural aspects of disaster management, and in target group-focused MHPSS action sheets for children and adolescents, older people, and refugees. Additionally, the OPSIC guidelines contain two action sheets on MHPSS preparedness in general (“general principles for MHPSS in disasters” and “key MHPSS principles for preparedness”) and several action sheets with specific preparedness topics (e.g., “social media”, “older people”). The OPSIC guidelines provide a set of “characteristics of best programming”, derived from expert interviews. According to experts, MHPSS preparedness should be based on/involve: (1) principles of latest research/guidelines; (2) stable funding throughout the response period; (3) a multidisciplinary preparedness consultancy group; (4) predefined follow up system and co-operation with mental health systems (e.g., to set up of referral routes); (5) access to volunteers; (6) structured training and support for staff and volunteers; (7) co-operation with other key organizations; and (8) a plan for installing information and resource centers and services for the affected.

The Impact guidelines are less detailed. Instead, they recommend to implement the guidelines in a holistic way by, “among others: (1) translating the guidelines into specific guidance, tools or protocols for distinct user groups and/or subtasks; (2) making the guidelines an integrated part of the preparation for disasters and crises (in planning, education, training and exercises), monodisciplinary and multidisciplinary, and by checking the level of preparedness periodically; (3) investing broadly in the motivation, capability and opportunity of user groups at different levels to adhere to the guidelines (e.g., by communicating the importance, by creating space in daily activities, by eliminating obstacles and by checking adherence periodically)”.

### 3.3. Conditions for Implementation and Preparedness

When going through the guidelines, there are clear indications that training, planning, mapping of vulnerabilities and service capacity, and other activities on behalf of preparation are thought to increase preparedness and are, in that sense, a condition for guideline implementation. Implementation and preparation are closely related concepts, entailing investments in guideline-based capacity building. Both concepts involve processes that require meeting certain criteria. Each of the guidelines acknowledges factors such as resources (financial and personnel), awareness, expertise, coordination, plans, and protocols. Although it might seem a condition sine qua non, financial resources (“money” in particular) are not receiving a great deal of explicit attention in the documents analyzed. The OPSIC guidelines speak of “stable funding”, the Red Cross guidelines encourage, following assessment, to develop “a comprehensive report outlining detailed recommendations for programme design, including the strategy, the time frame, and the financial and personnel resources required”.

Sometimes the term “leadership” is used directly, such as in the IASC guidelines, where leadership of mental health professionals is considered a prerequisite for the implementation of clinical interventions. The OPSIC guidelines highlight the importance of leadership several times; however, apart from something “staff and volunteers should [effectively] have”, “lead by example and spearhead the dialogue”, and “set the tone in public debate on tolerance and non-discrimination”, a clear description of what leadership entails is not given. Obviously, the implementation of the MHPSS guidance itself can be seen as an all-encompassing leadership task. Take, for example, the recommendation in the Red Cross guidelines to invest in the “supervision of implementation” of MHPSS programs: deploy someone to oversee MHPSS design and implementation, a delegate present for a longer time period.

In the Impact guidelines, implementation factors are discussed explicitly, referring to dozens of factors that are confirmed to be linked to the success (or failure) of implementing changes and the motivation, capability, and opportunity of people involved, across domains and levels. The distinction in motivation, capability, and opportunity as behavioral determinants, based on the “behavioural change wheel” (or “COM-B-model”) [61], covers an array of conditions that help or hinder the target group to adhere to the guidelines. The Impact guidelines encourage the target group to invest in these conditions and stress that professionals are, because of what professionalism entails, obliged to optimize their own motivation, capability, and opportunity, being more or less facilitated by their affiliated organizations.

## 4. Discussion

### 4.1. Main Findings

The objectives for this comparative content analysis of MHPSS guidelines were to explore basic characteristics of the guidelines, to disentangle the nature of the guidance, and to examine implementation or preparedness conditions recognized in the documents.

#### 4.1.1. Characteristics of the Guidelines

An exploration of basic characteristics of the guidelines revealed that the guidelines have been developed for high-income as well as low-income application settings, with Red Cross focusing more on low-income countries, Impact more on high-income, and IASC and OPSIC on both. Three of the guidelines are published in English, one in Dutch. They all work with comparable MHPSS definitions, describing services that, without exception, seek to promote mental health and well-being and, in some cases, with the aim to reduce or prevent psychopathology; the first can be interpreted as a “best effort obligation”, and the second as a “result obligation”. Although different in length and structure, the objectives of the guidelines are to provide information and tools to structure the planning and delivery of MHPSS in crisis situations, focusing on aiding user groups of authorities, professionals, and volunteers. The target groups are comprised of disaster-affected populations, and the degree of differentiation in vulnerable target groups varies.

#### 4.1.2. Nature of the Guidance: The Common Ground in the MHPSS Knowledge Base

The nature of the guidance in the documents we reviewed is remarkably similar. In each of the guidelines, language is used that reflects an orientation on the potential strengths and weaknesses of MHPSS target populations on the one hand (e.g., resilience, vulnerabilities, risks, needs, problems, psychopathology), and preventive and reactive responses on the other. This language (see Table 2) is complemented by several models that, in combination, tell a story with principles to pursue in relation to the (lack of) capacity of individuals to cope with adversity in more or less well-equipped support settings (informal and formal social networks; community ties devolving into professional health care systems), urging user groups to remain critical about one’s attitude towards target groups (Figure 1). Additionally, the guidelines recommend dozens of MHPSS measures and interventions that specify what basic aid, information, emotional and social support, practical support, and health care under the umbrella of MHPSS entails. When it comes to health care, and, more notably, to anticipating psychopathology, the guideline developers seem to apply the precautionary principle and are careful with recommendations on early therapeutic or preventive interventions. Apparently, the inconclusive state of the scientific evidence on effectiveness is interpreted rather conservatively: better safe than sorry.

Each of the guidelines encourages its user groups to take monitoring and evaluation seriously and urges investment in implementation and preparedness to ensure the practical uptake of its content. Furthermore, users of the guidelines are reminded repeatedly to act in a context-sensitive way when planning and implementing services for beneficiaries. The IASC, OPSIC, and Red Cross guidelines also consider cultural sensitivity, ethics, and human rights. Overall, despite differences in accents and level of detail in the guidance provided, our analysis points at a “common ground” within the MHPSS knowledge base embedded in available guidelines.

#### 4.1.3. Conditions for Implementation and Preparedness

Implementation and preparedness are closely related concepts in the guidelines. Activities during the preparedness phase (e.g., training, planning, mapping) are seen as factors contributing to guideline implementation in the acute phase and recovery phase. Each of the documents recognizes the availability of resources (financial and personnel), awareness, expertise, coordination, plans, and protocols as relevant conditions, and urges the continued investment in their quantity and quality.

### 4.2. Implications for Disaster Risk Reduction

One way to determine the value of MHPSS guidelines for DRR is to examine whether the documents are used to deal with threats for mental health and well-being when crisis situations take place. In the Netherlands, the Impact guidelines were used, for instance, to plan, implement, and evaluate the coordinated service delivery for bereaved families after the MH17 airplane crash in Ukraine in 2014, and to structure the public mental health response to the COVID-19 pandemic [62,63]. Internationally, specific MHPSS guidance on behalf of general populations affected by the COVID-19 pandemic was formulated based on, among others, the IASC guidelines [64,65]. In a literature review conducted during the COVID-19 pandemic, the OPSIC guidelines were referred to as a standard to organize psychosocial services for mental health professionals in pandemics [66].

However, from a long-term perspective, it is important to incorporate MHPSS guidance in strategies to reduce disaster mental health risks in a broader sense, not limited to certain events or hazards. This implies that MHPSS knowledge should be integrated in DRR activities.

#### 4.2.1. Using the Guidance to Overcome Integration Challenges

In the introduction, we described three challenges for the integration, identified by Gray and colleagues [20]. The first challenge was linked to the lacuna of an overarching framework, definition or a consensus-based model for discussing MHPSS components of DRR, two fields largely discussed in isolation. We can agree with the latter, though we are more optimistic about the availability of a useful framework. The common ground, observable in existing guidelines, can, at best, serve as this framework and, at minimum, as a skeleton or foundation to realize a unifying narrative. This brings us to the next challenge: the lack of a shared idea of the activities (including best practices for stakeholder collaboration) constituting MHPSS–DRR integration. We agree that integration cannot take place without stakeholder engagement and co-learning. Moreover, good examples of how to approach and facilitate an effective dialogue contributing to risk reduction are certainly needed. Our suggestion would be to follow the train of thought visualized in Figure 1 and to collect data on promising best practices and how they function in different DRR contexts. Co-learning across domains, driven by MHPSS guidance, contributing to shared knowledge about vulnerabilities and potential solutions, and the uptake of this knowledge in policy and practice, could be a core pillar of the integration of MHPSS approaches into DRR. Next, and this is connected to the third challenge, experiments with different participatory knowledge development and valorization processes, followed by a robust evaluation are most likely a crucial step to achieve evidence-based or consensus-driven definitions and guidelines for the integration of MHPSS and DRR, as recommended by Gray et al. [20]. Such guidelines, arguably leading to a better positioning of MHPSS in DRR programming, can be seen as guidelines to implement MHPSS guidelines on behalf of risk reduction. In our view, the content of these “guideline implementation guidelines” should build further upon the international MHPSS knowledge base and serve as part and parcel of the dialectic process shown in Figure 1.

#### 4.2.2. Cross-Level Integration: Translating Universal Guidance to Specific Contexts

What we did not contemplate so far is that risk reduction activities can vary in proactiveness. Obviously, it is important to learn from earlier successes and failures solutions that might be worth considering again or pitfalls to avoid. Insights like these are welcome as building blocks for guideline development and preparedness planning, and to assist decision-making when new disasters occur. A higher level of proactiveness is achieved when preventive measures are taken beforehand to minimize risk. After all, many of the risks and vulnerabilities discovered by disaster mental health research are often pre-existing vulnerabilities; disasters expose and utilize underlying demographic, socioeconomic, and structural vulnerabilities in human systems. Whether MHPSS and DRR are approached as separate or integrated fields, prevention is a relevant theme for both of them; MHPSS guidance encourages authorities and service providers to address risks and vulnerabilities in the wake of a crisis, as it fits within the logic of DRR to start as early as realistically possible, preferably before the crisis.

Additionally, whether distinct or integrated fields, MHPSS and DRR need to formulate evidence-based guidance that is generic and practically applicable at the same time. In the accompanying review of MHPSS guidelines, substantial variation in “implementability” and room for improvement was found between the guidelines [40]. The reality is that the implementation context of MHPSS guidelines is not homogenous. Demographic, cultural, and socioeconomic variation in contexts confront target groups with the need to reinterpret the general content of MHPSS guidelines and tailor it to national and local service delivery systems [67].

This contextual “reinvention exercise” is relevant to each of the models depicted in Figure 2. From a resilience or vulnerability perspective (Model 1), people’s susceptibility to being harmed or their ability to cope, benefitting from relative support in their own supportive environment, differs within and between subgroups and populations, even regionally, and changes over time [68,69,70]. Resilience cannot be seen separately from cultural and socioeconomic conditions at the individual, community, and societal level. It varies, independently from (or in combination with) exposure to disasters, war, and other potentially traumatic events [2,51,71,72]. Likewise, the guidance must be operationalized locally in relation to available capacity and technology to (a) bring the Five Essential Principles into practice for people in need (Model 2), and (b) to organize and provide supportive services in the layered Pyramid Model (Model 3) and along the Circle Model (Model 4). Given that expectations of beneficiaries and the service capacity will differ between communities and populations, depending on the moment of time in the disaster timeline and the broader geographical disaster vulnerability context [67], evaluations of service delivery quality (Model 5), will be difficult to compare. It is important, nonetheless, to ensure that perspectives of all stakeholders are included in the planning, implementation, and evaluation of MHPSS services, and to use evaluation results for improvement of the response (Model 6), as well as prevention and preparedness efforts.

The disaster risk reduction mechanism functions even better when local insights and potential lessons are included in MHPSS knowledge development processes at the national or even global level. In that case, the challenge works the other way around. In a nutshell: *From global to local*, it is important to enrich general guidance with information that makes it applicable in contexts with unique particularities, while *from local to global*, some of these particularities need to be translated and unglued from contextual constraints, resulting in more universally valid insights. As such, in the chain of MHPSS knowledge development and uptake on behalf of DRR, combining activities in two focal areas, as shown in Figure 1, is in fact a multi-level process. At the start of each series of steps, information on needs, problems, risks, and vulnerabilities (Focal area 1) or on adequate measures and interventions (Focal area 2) can come from different levels. Eventually, when knowledge is produced that is primarily custom-made for MHPSS–DRR activities at a specific level, parts of this knowledge might also be relevant for activities at other levels. We tend to go even further. Can MHPSS–DRR integration be considered complete if it is not coupled with an effective cross-level MHPSS knowledge exchange feeding the policy and practice of DRR?

#### 4.2.3. Closing the Cycle: Disaster Risk Reduction through Learning

Despite a decade of important progress in strengthening the MHPSS evidence base, available evaluations still insufficiently allow us to understand how to reduce mental health risks in the short and long term for people confronted with adversity [67,73,74]. The MHPSS guidelines we assessed tell a plausible story of how needs, problems, and risks should be followed up, but they run short of providing empirical evidence on how the common ground of knowledge is effective in its mitigation promise. When the guidelines are implemented in the prescribed manner, measures and interventions applied in response to an event will be evaluated. Monitoring and evaluation offer an opportunity for involved stakeholders to determine whether the response contributed to the reduction of the initial problem. If this is the case, and if the methodology and findings are disclosed in a transparent way and properly translated, others—at the same or different levels in the multi-level DRR process—can learn from these evaluations as well. This type of learning requires investments in prevention and preparedness that lower the chance of repetition and strengthen the response capacity for new difficult times. The problem with these investments is that they require a decision on the current allocation of scarce resources based on future uncertainty. The paradox of DRR is that relatively large costs for issues, which today seem less urgent in the light of obvious acute problems, overshadow vaguer (and by chance larger) long-term costs of inaction and consecutive disasters.

## 5. Conclusions

In this contribution, which fits within a growing literature seeking to understand and stimulate the integration of MHPSS into DRR, we started with sketching a conceptual framework for multi-sectoral dialogue, linking collaborative problem identification to co-learning in the development and application of solutions. We examined the content of existing high-quality MHPSS guidelines, forming the heart of the international knowledge base, to learn about definitions, focus, user and target groups, terminology, models, recommended measures and interventions, and recognized conditions for implementation and preparedness. Based on the analysis, we conclude that the MHPSS knowledge base is rich. Existing guidelines have a lot to offer in terms of meaningful content for DRR. This knowledge base is, at the same time—and this is somewhat surprising—fairly modestly reflected upon in integrative literature. We understand challenges identified for the integration of the MHPSS and DRR fields, but believe the available common ground present in the combined guidance provides sufficient opportunity to overcome these challenges. However, regular updates of guidelines and new studies to correct flaws, fill in gaps, and explore new themes must be welcomed. Progress in MHPSS–DRR integration may require something else: the *cycle* of defining risks, problems, and vulnerabilities, and subsequently, designing and implementing solutions, and finally, evaluating how the solutions that lead to improvement needs to be completed at the local, national, regional, and global levels, again and again. In each cycle, and at every level, generic challenges and contextual idiosyncrasies play a role, linked to problems and solutions, that might only be considered and addressed effectively when stakeholders (including beneficiaries) with contextual knowledge are engaged in the process. In order to align progress across levels rooted in the MHPSS knowledge base, it helps if some of these stakeholders are able to oversee how local progress serves DRR objectives at higher levels and vice versa. A noteworthy challenge in this respect is that we should be critical about the merits of the information produced and shared. Generic guidance, unhelpful at the grassroot level, albeit internationally agreed upon, or local lessons not contributing to DRR elsewhere, is of marginal value in the light of dissemination. Lastly, how the content of guidelines works out in practice in specific disaster settings is still poorly evaluated. Without improvement, it will continue to complicate our ability to test the practical worth of MHPSS guidance, whether assessed at itself or seen in the light of DRR.

## Figures and Tables

**Table 1:** MHPSS guidelines: definitions, purpose, user groups, and target groups.

Guidelines	MHPSS Definition	Purpose	User Groups	Target Groups
IASC, 2007	A composite term to describe any type of local or outside support that aims to protect or promote psychosocial well-being and/or prevent or treat mental disorder.	Enable humanitarian actors and communities to plan, establish, and coordinate a set of minimum multi-sectoral responses to protect and improve people’s mental health and psychosocial well-being in the midst of an emergency. Additionally, list concrete strategies for mental health and psychosocial support to be considered, mainly before and after the acute emergency phase.	Humanitarian actors including community-based organizations, government authorities, United Nations organizations, NGOs, and donors operating in emergency settings at the local, national, and international levels.	Young children and care givers; people with severe physical, neurological, or mental disabilities or disorders; women (e.g., pregnant women, (single) mothers, widows, and, in some cultures, unmarried adult women and teenage girls); men (e.g., ex-combatants, young men at risk of detention, abduction, or being targets of violence); people exposed to extremely stressful events/trauma; people experiencing severe social stigma; people at specific risk of human rights violations (e.g., political activists, ethnic or linguistic minorities); helpers and staff; refugees; internally displaced persons; migrants in irregular situations; elderly; people in institutions (e.g., orphans, elderly, people with neurological/mental disabilities or disorders); extremely poor people.
Impact, 2014	All support and care focused on the psychological well-being and the health of people affected, provided by the user group of the guideline, in the acute and recovery phase, for the individual as well as groups.	Offer a frame of reference and a tool for providing MHPSS. Facilitate quality improvement, optimize MHPSS, and professionalize the user group of the guidelines.	Governments and public services, aid workers (professionals and volunteers), and their organizations.	All relevant groups within the affected population including people with (a history of) psychiatric problems; adolescents; children; mothers with young children; ethnic minorities; migrants; refugees; people who previously experienced potentially shocking events; people affected with limited access to social support and resources; affected staff members.
OPSIC, 2016	Providing a humanitarian response in ways that are beneficial to the mental health and psychosocial well-being of the beneficiaries.	Point users to relevant guidelines, resources, and tools for planning and implementing MHPSS programs at all phases of response and in all types of disasters, and with all possible target groups.	Decision-makers, crisis managers (including incident command and psychosocial crisis managers), mental health professionals in multi-agency coordination groups, and practitioners.	All relevant groups within the affected population; children and adolescents; elderly; disabled persons; refugees; helpers (staff and volunteers); people with severe mental disorders; marginalized people; women and girls.
Red Cross, 2018	*Mental health:* used to denote psychological well-being. Mental health interventions aim to improve psychological well-being by reducing levels of psychological distress, improving daily functioning, and ensuring effective coping strategies. *Psychosocial:* used to describe the interconnection between the individual (i.e., a person’s ‘psyche’) and their environment, interpersonal relationships, community, and/or culture (i.e., their social context). *Psychosocial support:* essential for maintaining good physical and mental health and provides an important coping mechanism for people during difficult times.	Encompass internationally recognized, evidence-based MHPSS standards and practices, combined with the expertise, experience, and views of mental health professionals who have worked in armed conflict and other violence. They are designed to be adapted and developed over time, and set out a framework of ethical principles, common definitions, and recommended procedures to be applied to the ICRC’s MHPSS activities.	Local, national, and international Red Cross organizations.	People affected by emergencies; victims of (sexual) violence; hospitalized weapon-wounded patients; families of victims/missing persons; helpers; people deprived of their liberty and former detainees; people with a physical or mental disability; children (e.g., separated from their families, associated with armed groups); elderly; marginalized social groups within the community.

**Table 2:** Frequency of Terminology in MHPSS guidelines.

	Term	IASC	Impact	OPSIC	Red Cross
Focal area 1	Resilience	9	35	197	5
	Vulnerable/Vulnerability/Vulnerabilities	26	11	71	35
	Risk/Risks (Risk factors)	133 (2)	17 (30)	209 (15)	19 (5)
	Needs	56	66	271	376
	Problem/Problems	102	84	80	57
	Disorder/Disorders	111	13	82	69
	Trauma/Traumatic	12 (20)	11 (10)	145 (138)	34 (20)
	PTSD *	11	14	211	2
Focal area 2	Prevent/Preventive/Prevention	75	17	194	28
	Assessment/Assessments	153	1	270	80
	Needs assessment/assessments	2	1	28	34
	Screening/Screened	4	11	50	5
	Monitoring	52	3	93	37
	Evaluation/Evaluations	36	28	115	19
	Monitoring and evaluation/evaluating	27	0	14	28
	Coordination	100	4	74	4
	Preparation/Prepare/Prepared/Preparedness	35	29	278	8
	Implementation	24	12	31	32
	Training	121	3	211	78
	Resources	147	7	268	57
	Conditions **	32	5	29	21
Focal areas 1 and 2	Circumstances	2	11	20	12
	Context/Contexts	40	25	84	18
	Phase/Phases	47	66	182	2
	Culturally appropriate/sensitive/specific	52	4	24	4
	Ethics/Ethical	29	0	63	15
	Human rights	91	0	38	3

* Post-traumatic stress disorder; ** Health conditions excluded. Dutch translation in Appendix A.

**Table 3:** Five Categories of Recommended Measures and Interventions.

Basic Aid	Information	Emotional and Social Support	Practical Support	Health Care
Shelter ^1,2,3,4^ Evacuation ^1,3^ Basic medical assistance and (continuation of) medication ^1,2,3,4^ Food and water (nutrition) ^1,2,3,4^ Hygiene/Sanitation ^1,2,3,4^ Family reunification ^1,2,3,4^ Host families ^1,2,3^ Replacement housing ^1,3,4^	*Content:* Psychoinformation/Psychoeducation ^1,2,3,4^ Sensitization to MHPSS issues ^2,3^ Information about the emergency situation, relief efforts, and status of the affected (e.g., family members) ^1,2,3,4^ *Methods:* Information meetings (e.g., for affected population, care givers) ^1,2,3,4^ Local/national referral information website/telephone services (e.g., also for other service providers incl. MHPSS workers) ^3,4^ Written leaflets ^1,2,3,4^ (Social) Media (e.g., radio, TV) ^1,2,3,4^	*Individual:* Psychological first aid (PFA) ^1,2,3,4^ Peer support ^1,2,3,4^ Support after (ambiguous) loss/grief ^1,2,3,4^ Office consultations (when home visits are not feasible) ^2^ *Community:* (Pre-)School interventions ^1,3^ Group-based interventions ^1,2,3^ *Not recommended: Psychological debriefing ^1,3,4^* Family-based interventions ^1,2,3^ Religious, cultural, and spiritual supports ^1,2,3,4^ *Collective:* Humanitarian assistance center/Reception center/One-stop shop (physical or virtual) ^3,4^ Telephone helpline ^3^ Commemorative activities ^2,3,4^	Provision of means of communication ^4^ Transport ^2,3,4^ Legal support and testimony services ^1,2,3,4^ Financial support ^2,3,4^ Compensation ^4^ Education ^1,2,3^ Rehabilitation services: e.g., employment services ^1,2,3^ Administrative support ^2,4^ Help with household chores ^4^ Family tracing services ^1,2,3^ Insurance policy/Reimbursement for MHPSS volunteers ^1,3^	Ensure access to more specialized (mental) health care ^1,2,3,4^ Mental health triage ^1,2,3,4^ Screening (risk and vulnerability factors) ^1,2,3,4^ Comprehensive mental health assessment (surveys based on cross-culturally validated standardized instruments) ^1,2,3,4^ Clinical counselling ^1,2,3,4^ *Therapy, treatment:* Psychotherapy ^1,2,3,4^ (e.g., Eye Movement Desensitization and Reprocessing (EMDR) ^3,4,^* and Cognitive behavioral therapy (CBT) ^3,4^) Interventions in case of substance use/addiction ^1,3,4^ Pharmacological treatment ^1,2,3^

^1^ IASC guidelines; ^2^ Red Cross guidelines; ^3^ OPSIC guidelines; ^4^ Impact guidelines; * Not recommended for acute stress disorder.

## Appendix A

**Table A1:** Dutch version of terminology used to fill Table 2.

Focal area 1	Veerkracht (17) Zelfredzaamheid (18)
	Kwetsbaar/Kwetsbaarheid/Kwetsbaarheden/Kwetsbare (11)
	Risico/Risico’s (17) Risicofactoren (30)
	Behoeften (66)
	Probleem/Problemen (84)
	Stoornis/Stoornissen (13)
	Trauma/Traumatisch (21)
	PTSS (14)
Focal area 2	Preventie/Preventief/Preventieve (17)
	[no translation for Assessment/Assessments] (-)
	Behoeftepeilingen (1)
	Screening (10) Screeningonderzoek (1)
	Monitor/Monitoring (3)
	Evaluatie/Evaluaties (28)
	Monitoring and evaluatie (0)
	Coördinatie/Coördinatietaak/Coördinatietaken (4)
	Preparatie (9) Voorbereiden/Voorbereiding (20)
	Implementatie (12)
	Training (3)
	Middelen (7)
	Condities (1) Randvoorwaarden (4)
Focal areas 1 and 2	Omstandigheden (11)
	Context (25)
	Fase/Fasen (66)
	Cultuurspecifiek/Cultuurspecifieke (4)
	Ethiek/Ethisch (0)
	(Mensen)rechten (0)
